# Optimal allocation of leaf epidermal area for gas exchange

**DOI:** 10.1111/nph.13929

**Published:** 2016-03-16

**Authors:** Hugo J. de Boer, Charles A. Price, Friederike Wagner‐Cremer, Stefan C. Dekker, Peter J. Franks, Erik J. Veneklaas

**Affiliations:** ^1^ Department of Environmental SciencesFaculty of GeosciencesUtrecht UniversityHeidelberglaan 23584 CSUtrechtthe Netherlands; ^2^ School of Plant BiologyThe University of Western Australia35 Stirling HighwayCrawleyWA6009Australia; ^3^ National Institute for Mathematical and Biological Synthesis (NIMBioS)University of Tennessee1122 Volunteer Blvd, Suite 106KnoxvilleTN37996‐3410USA; ^4^ Department of Physical GeographyFaculty of GeosciencesUtrecht UniversityHeidelberglaan 23584 CSUtrechtthe Netherlands; ^5^ The University of SydneyFaculty of Agriculture and EnvironmentSydneyNSW2006Australia

**Keywords:** allometry, evolution, guard cell, leaf gas exchange, leaf morphology, stomata

## Abstract

- A long‐standing research focus in phytology has been to understand how plants allocate leaf epidermal space to stomata in order to achieve an economic balance between the plant's carbon needs and water use. Here, we present a quantitative theoretical framework to predict allometric relationships between morphological stomatal traits in relation to leaf gas exchange and the required allocation of epidermal area to stomata.
- Our theoretical framework was derived from first principles of diffusion and geometry based on the hypothesis that selection for higher anatomical maximum stomatal conductance (*g*_smax_) involves a trade‐off to minimize the fraction of the epidermis that is allocated to stomata. Predicted allometric relationships between stomatal traits were tested with a comprehensive compilation of published and unpublished data on 1057 species from all major clades.
- In support of our theoretical framework, stomatal traits of this phylogenetically diverse sample reflect spatially optimal allometry that minimizes investment in the allocation of epidermal area when plants evolve towards higher *g*_smax_.
- Our results specifically highlight that the stomatal morphology of angiosperms evolved along spatially optimal allometric relationships. We propose that the resulting wide range of viable stomatal trait combinations equips angiosperms with developmental and evolutionary flexibility in leaf gas exchange unrivalled by gymnosperms and pteridophytes.

## Abstract 2

See also the Commentary on this article by Lawson & McElwain, **210**: 1149–1151.

## Introduction

The colonization of land by terrestrial plants was enabled by the evolution of specialized pores (stomata) on the leaf epidermis that regulate the exchange of water vapour and CO_2_ between the leaf interior and the atmosphere (Kenrick & Crane, 1997). Crucially, stomata solved the functional dilemma of facilitating CO_2_ diffusion into the leaf for photosynthesis while also limiting the outward diffusion of water vapour (Nobel, 1999). However, this innovation came with one large constraint: the fraction of the epidermis that is allocated to stomata critically determines the benefit they offer. To function properly stomata need to be adequately spaced (Franks & Farquhar, 2007), but to facilitate the highest rates of leaf gas exchange the stomata also need to be present in sufficiently high numbers on the leaf surface (Parlange & Waggoner, 1970). Therefore, both insufficient and excessive investment in stomata incur disadvantages that ultimately influence plant productivity, competition and survival (Vatén & Bergmann, 2012). Despite this fundamental importance, a general theory to explain how plants allocate leaf epidermal area to stomata is lacking. Here, we propose a quantitative theoretical framework to describe the allocation of leaf epidermal area to stomata based on the allometric relationship between morphological stomatal traits and the resulting gas exchange capacity of the epidermis.

**Figure 1:**
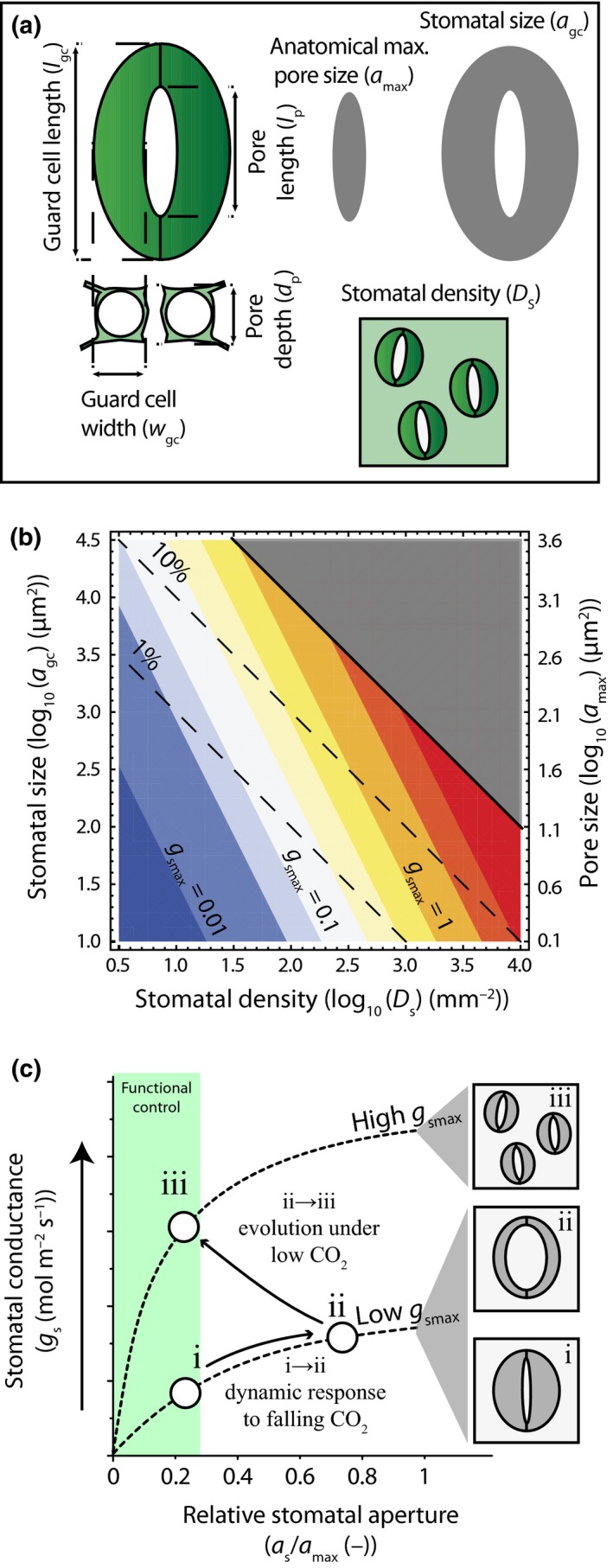
Theoretical relationships between morphological stomatal traits and leaf gas exchange. (a) Schematized stomatal morphology following Lawson *et al*. (1998) and the specific stomatal traits considered in this study. Stomatal size (*a*_gc_) and anatomical maximum pore size (*a*_max_) are indicated with grey shapes. (b) Theoretical relationship between the leaf epidermal area fraction allocated to stomata (*f*_gc_) (dashed lines and expressed as %) and the anatomical maximum stomatal conductance (*g*_smax_) (shaded colours and expressed as mol m^−2^ s^−1^). The *f*_gc_ is expressed as a function of log_10_‐transformed values of stomatal density (*D*_s_) and *a*_gc_, whereas *g*_smax_ is expressed as a function of log_10_‐transformed values of *D*_s_ and *a*_max_ (plotted on the 2^nd^ *y*‐axis). A constant ratio between *a*_max_ and *a*_gc_ of 0.12 (Franks & Beerling, 2009) is assumed for plotting, although this ratio may change depending on stomatal morphology (Franks *et al*., 2014). (c) Schematic response of stomatal conductance (*g*_s_) to a short‐term decrease in atmospheric CO_2_ (Farquhar & Sharkey, 1982) before a long‐term decrease in atmospheric CO_2_ (i → ii), and changes in *a*_gc_ and *D*_s_ during a long‐term decrease in atmospheric CO_2_ concentration (Franks & Beerling, 2009) that restore functional control on *g*_s_ by increasing *g*_smax_ (Franks *et al*., 2012b) (ii → iii). The relative stomatal aperture is indicated by the ratio of stomatal aperture (*∂*_s_) to *∂*_max_. The green area denotes the region where guard cells have most functional control on *g*_s_.

The basic morphology of stomata consists of two guard cells that regulate the aperture of a central diffusion pore. A graphical representation of the morphological stomatal traits considered in this study is shown in Fig. 1(a). Stomata may occur on both the upper and lower leaf surface (amphistomaty), or on one leaf surface, which is typically the lower surface (hypostomaty). With the formation of each leaf, the average fraction of the leaf epidermis that is allocated to stomata (*f*_gc_) is determined by the average size of the guard cell pair (*a*_gc_) and average stomatal density (*D*_s_) (Fig. 1b). Together with the size of the stomatal pore at its anatomical maximum aperture (*a*_max_), these traits determine the anatomical maximum stomatal conductance (*g*_smax_) of the leaf epidermis. Plants regulate leaf gas exchange dynamically by adjusting stomatal conductance (*g*_s_) over a range between near‐zero and *g*_smax_ by opening and closing the stomatal pore at a time scale of minutes to hours (Farquhar & Sharkey, 1982). These aperture changes may occur through a combination of (passive) responses to leaf water status or (active) responses to abscisic acid (McAdam & Brodribb, 2012, 2014). Although *g*_s_ can attain values near *g*_smax_ under laboratory conditions with low CO_2_ and high humidity (Dow *et al*., 2014), the operational stomatal conductance is relatively constant around a ratio *g*_s_ : *g*_smax_ of 0.2–0.25 under typical growth conditions (McElwain *et al*., 2015). This ratio corresponds to the region where changes in guard cell turgor pressure have most effective control on stomatal aperture (Franks *et al*., 2012b). Adjustment of *g*_smax_ under sustained environmental pressure may therefore confer a functional advantage because it allows more efficient dynamic control on leaf gas exchange under typical growth conditions. The clearest example of this adaptation is that plants respond to prolonged CO_2_ changes by adjusting *g*_smax_ via changes in stomatal densities (Woodward, 1987) and sizes (Franks & Beerling, 2009) in line with a short‐term (dynamic) response to CO_2_ (Franks *et al*., 2013) (Fig. 1c). These adaptations may occur through evolution (Franks & Beerling, 2009) or phenotypic changes (de Boer *et al*., 2011; Lammertsma *et al*., 2011), whereby the latter appear most pronounced in species with limited active stomatal responses to changes in atmospheric CO_2_ (Haworth *et al*., 2013).

Owing to the close relationship between plant productivity, leaf gas exchange and *g*_smax_ (McElwain *et al*., 2015), the density and size of stomata on every leaf, including the proportion of leaf surface they occupy, represents a critical investment in the functional economics of the plant. However, the potential for plants to increase *g*_smax_ is fundamentally constrained by available space on the leaf epidermis that can viably be allocated to stomata. Three mechanisms have been proposed that relate this spatial constraint to plant function. First, too close spacing of neighbouring stomata may hamper effective opening and closing responses because guard cell movements depend partly on the mechanical advantage of the subsidiary cells (Franks & Farquhar, 2007). Second, as stomata are costly in terms of the energy needed for their operation and maintenance (Assmann & Zeiger, 1987), excess stomata may negatively affect the leaf carbon balance. Third, an increase in stomatal density without concurrent size reduction places stomata closer together and leads to increased interference between the diffusion shells of neighbouring stomata (Lehmann & Or, 2015). From this perspective, evolution pressure on stomatal morphology reflects a trade‐off between the benefit of increasing leaf gas exchange and the cost associated with increasing the allocation of leaf epidermal area to stomata. Understanding this trade‐off remains relevant for acclimation and adaptation of modern plants (Franks *et al*., 2013) because, although current atmospheric CO_2_ levels are relatively low compared with those that have occurred since the Cretaceous (Fletcher *et al*., 2008), CO_2_ levels have increased dramatically in the last century and are expected to increase further this century (van Vuuren *et al*., 2011).

Despite a long history of research in this field, a quantitative theory to explain the allometric relationships between morphological stomatal traits is still lacking. Here we derive and test a quantitative theoretical framework that predicts two key allometric relationships between morphological stomatal traits. The first relationship entails the scaling between stomatal sizes *a*_gc_ and stomatal densities *D*_s_. The second entails the scaling between anatomical maximum pore sizes *a*_max_ and stomatal sizes *a*_gc_. These relationships are derived from generic constraints related to the investment of leaf epidermal area to stomata and the resulting *g*_smax_. We test the predicted allometric relationships with a comprehensive compilation of published and unpublished data on the stomatal traits of 1057 species from 156 families that include all major clades and reflect a global range of environments. Our empirical analyses are performed at tree levels of detail. First, we analyse allometric relationships between the morphological stomatal traits of a phylogenetically diverse species group. Secondly, we differentiate between the evolutionary distinct groups of pteridophyte, gymnosperm and angiosperm species. Finally, we analyse a selection of amphistomatous species that, by allocating space on both leaf surfaces to stomata, may reflect an exception to the proposed spatial constraint on stomatal size–density combinations.

## Description

### Theoretical framework

The theoretical framework presented here is developed based on two premises. The first premise is that evolution of stomatal density together with the size of the guard cells and pores principally reflects selection pressure to realize the benefit of increased *g*_smax_ (Franks & Beerling, 2009; McElwain *et al*., 2015). The second premise is that there is a cost associated with increasing the fractional stomatal cover of the leaf epidermis (*f*_gc_). Hence, *f*_gc_ is considered a proxy for the combined costs associated with the operation and maintenance of the stomata (Edwards *et al*., 1976; Assmann & Zeiger, 1987; Franks & Farquhar, 2007). Our hypothesis entails that the evolution of morphological stomatal traits results in an increase in *g*_smax_ (the benefit) and a simultaneous decrease in *f*_gc_ (the cost). From our framework we can derive testable predictions by expressing *g*_smax_ and *f*_gc_ in terms of (observable) allometric relationships between the stomatal density *D*_s_, the size of the guard cell pair *a*_gc_ and the anatomical maximum pore size *a*_max_.

Here, the *f*_gc_ was calculated as the product of stomatal density and stomatal size:

(Eqn 1) $$f_{\mathrm{gc}}=D_{s}\;\;\cdot \;\;a_{\mathrm{gc}}$$

The *g*_smax_ was calculated from the principles of diffusion (Franks & Farquhar, 2001; Franks & Beerling, 2009):

(Eqn 2) $$g_{s\;\max}=\frac{D_{s}\;\;\cdot \;\;a_{\max}\frac{d_{H_{2}O}}{w_{v}}}{d_{p}+\frac{\pi }{2}\sqrt{a_{\max}/\pi }}$$

(d_H2O_, diffusivity of water vapour in air; *w*_*v*_ *,* molar volume of air normalized to 25°C; *d*_p_, depth of the stomatal pore.) For our theoretical analyses of *g*_smax_, *d*_p_ is assumed equal to the cross‐sectional diameter, or width of the guard cell (*w*_gc_) (Franks & Farquhar, 2007) and retains a constant ratio (*r*_wl_) with guard cell length (*l*_gc_) so that *r*_wl_ = *w*_gc_/*l*_gc_ ≈ 0.36 across species (Supporting Information Fig. S1). This allows us to express pore depth as: $d_{p}=\sqrt{a_{\mathrm{gc}}\;\;\cdot \;\;r_{\mathrm{wl}}\;\;\cdot \;\frac{2}{\pi }}$.

In order to derive testable predictions from our hypothesis, we represent the allometric relationship between sizes of the guard cell pairs and the stomatal density densities as:

(Eqn 3) $$a_{\mathrm{gc}}=b_{s}\;\;\cdot \;\;{D_{s}}^{S}$$

(*b*_s_, offset of the scaling relationship; *S*, scaling exponent.)

The values of *g*_smax_ and *f*_gc_ are linked via the allometric relationship between the anatomical maximum pore size and stomatal size:

(Eqn 4) $$a_{\max}=b_{p}\;\;\cdot \;\;{a_{\mathrm{gc}}}^{P}$$

(*b*_p_, offset of the scaling relationship; *P*, scaling exponent.)

By inserting the allometric relationships between *a*_gc_ and *D*_s_ Eqn 3 and between *a*_max_ and *a*_gc_ Eqn 4 into Eqns 1 and 2 we develop a mathematical cost–benefit expression of how *g*_smax_ changes relative to *f*_gc_ depending on combined changes in stomatal morphology and stomatal density (see Eqns 8–13 in Methods S1 and Notes S1 for details). This expression quantifies the change in *g*_smax_ resulting from changes in stomatal density and associated changes in stomatal morphology (∂ *g*_smax_/ ∂ *D*_s_), relative to the corresponding change in *f*_gc_ (∂ *f*_gc_/ ∂ *D*_s_), which we term the marginal ratio Λ:

(Eqn 5) $$\Lambda =\frac{\partial g_{s\;\max}/\partial D_{s}}{\partial f_{\mathrm{gc}}/\partial D_{s}}$$

**Figure 2:**
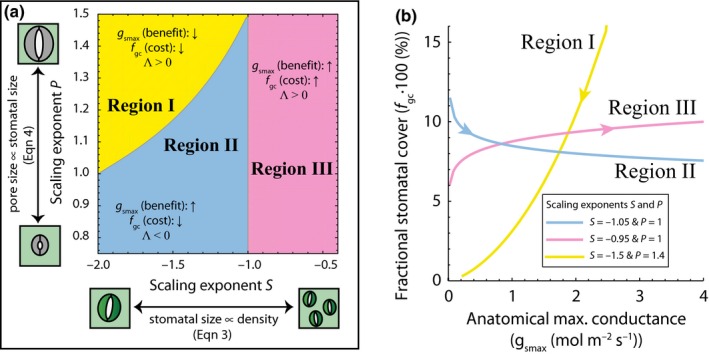
Theoretical framework on optimal allocation of leaf epidermal area for gas exchange. (a) Sign of the marginal ratio Λ Eqn 5, which represents the marginal change in *g*_smax_ under a change in stomatal density ($\partial g_{s\max}/\partial D_{s}$), relative to the corresponding marginal change in *f*_gc_ (∂ *f*_gc_/ ∂ *D*_s_), as a function of the exponents *S* and *P* in the allometric relationships between *D*_s_ and *a*_gc_ Eqn 3, and between *a*_gc_ and *a*_max_ Eqn 4, respectively. In regions (I) and (III), an increase in *D*_s_ leads to same‐signed changes of *g*_smax_ and *f*_gc_ (Λ > 0), whereas in region (II) an increase in *g*_smax_ is tied to a decrease in *f*_gc_ (Λ < 0). (b) Relationships between *g*_smax_ and *f*_gc_ depending on the scaling exponents *S* and *P*. The three lines correspond to the regions in (a). Arrows indicate the direction of change resulting from an increase in *D*_s_.

The full mathematical expression of Λ is difficult to interpret on its own and depends on the allometric relationships given by Eqns 3 and 4, principally the exponents *S* and *P*. Hence, we explored a range of realistic values for these exponents to characterize the behaviour of Eqn 5 This behaviour is characterized by the sign of Λ that allows us to recognize three regions which reflect different combinations of increasing/decreasing *f*_gc_ (cost) and *g*_smax_ (benefit) as a function of the exponents *S* and *P* (Fig. 2a). Based on this behaviour of Eqn 5, our framework yields two testable predictions. The first prediction requires that *f*_gc_ (the cost) should not increase when *D*_s_ increases. This prediction sets the upper bound on the exponent *S *≤* *−1, which is valid for region (I) and region (II) in Fig. 2(a). In these regions, the increase in *D*_s_ is tied to a decrease in *a*_gc_ in such a way that *f*_gc_ (calculated as *D*_s_·*a*_gc_, cf. Eqn 1 remains constant or decreases. The second prediction requires *g*_smax_ (the benefit) to increase when *D*_s_ increases. This occurs when combinations of scaling exponents *S* and *P* fall in regions (II) and (III) in Fig. 2(a). To satisfy both predictions, which yields Λ < 0, the combination of exponents *S* and *P* should therefore fall in region (II) in Fig. 2(a). Only this specific combination of exponents *S* and *P* results in an increase in *g*_smax_ and simultaneous decrease in *f*_gc_ (Fig. 2b). Our physiological interpretation of this situation is that the benefit of increasing *g*_smax_ is combined with a reduction in the cost associated with *f*_gc_.

### Data acquisition

In order to test our hypothesis, we compiled previously published and unpublished data on morphological stomatal traits of 1057 species from 156 families that include all major clades and reflect a wide range of environments. We searched the literature by querying the search engines of Scopus and Google scholar with the following search terms: ‘stomatal sizes’, ‘stomatal densities’, ‘guard cell dimensions’, ‘stomatal size’, ‘pore size’, ‘stomatal morphology’ and ‘cuticle morphology’. We specifically selected studies that included multiple measurements from single species, multiple species from a single family or multiple species from a single environment. References to the 50 studies included in the compiled dataset are presented in Table S1. From the selected studies we extracted data on stomatal density *D*_s_ in combination with the size of the guard cell pair *a*_gc_, guard cell length *l*_gc_, guard cell width *w*_gc_, stomatal pore length (*l*_p_) and pore depth *d*_p_. Typically, stomatal density was reported in literature for the abaxial (lower) leaf side assuming that the leaves are hypostomatous. In those situations, we reported stomatal density as an average density on the abaxial epidermis only. If stomatal density was provided for both leaf sides (with a stomatal ratio > 0.05) the species was flagged as being amphistomatous. To facilitate comparison with hypostomatous species and warrant consistency across all data, the stomatal densities of amphistomatous species reported here reflect the average density on the abaxial and adaxial epidermises. If the size of the guard cell pair *a*_gc_ was reported without reference to the guard cell dimensions *l*_gc_ and *w*_gc_, we used the reported value. Otherwise, the size of the guard cell pair was calculated as:

(Eqn 6) $$a_{\mathrm{gc}}=\frac{\pi }{2}\;\cdot \;\;l_{\mathrm{gc}}\;\;\cdot \;\;w_{\mathrm{gc}}$$

The pore size at its anatomical maximum aperture *a*_max_ was calculated from the reported pore length as:

(Eqn 7) $$a_{\max}=\frac{\pi }{4}f_{\mathrm{lw}}\;\;\cdot \;\;{l_{p}}^{2}$$

(*f*_lw_, area fraction of a circle with diameter *l*_p_ that is occupied by *a*_max_). For *f*_lw_ we differentiated the basic stomatal morphologies following Franks *et al*. (2014), where *f*_lw_ ranges between 0.4 and 1 (Table S2). For the species *Oplismenus hirtellus*,* Populus gileadensis* and *Tilia americana* we found *a*_max_ > *a*_gc_, which we deemed unrealistic. Owing to the relatively large uncertainty in measuring the length of the stomatal pore (Lawson *et al*., 1998) we excluded these values of *a*_max_ from our analyses. We note that this did not significantly alter the observed allometric relationships.

In addition to the published data, we included unpublished measurements of the stomatal traits of 43 species (Tables S1, S3). These measurements were taken by separating the cuticle through maceration of leaf fragments in 5–10% sodium hypochlorite (NaClO). The removed cuticle was dyed with safranin, and mounted in glycerine jelly on glass slides. Cuticles were analysed using optical microscopes and analysis software. Stomatal densities were determined by counting the number of stomata in a minimum of 10 fields of view per leaf. Stomatal dimensions were measured on a minimum of 10 stomata on each leaf.

### Data analyses and statistics

Species names as originally reported in the published literature were updated to the latest convention through the Taxonomic Name Resolution Service (Boyle *et al*., 2013). When multiple observations from a single species were available we used the arithmetic mean to calculate a species average trait value. If multiple averages for a single species were available from different sources, the grand mean was used as the species average. A compilation of all species averaged data on *D*_s_, *a*_gc_ and *a*_max_ is provided in Table S3.

We constructed a phylogenetic tree of the taxa included in our dataset with the Phylocom v.4.2 software (Webb *et al*., 2008) by using the APG III‐derived megatree R20120829 (Stevens, 2012). The nodes of the resulting phylogenetic tree were dated using the bladj function of Phylocom based on the dating of Wikström *et al*. (2001). The phylogeny of all species included in the compiled dataset is shown in Fig. S2. Based on this phylogeny we calculated the phylogenetically independent contrasts (PICs) (Felsenstein, 1985) of the traits considered using the R package Phytools (Revell, 2012). Branch lengths were transformed logarithmically to remove any relationship with the standardized contrasts. The phylogenetic signal in each trait was assessed with Blomberg *et al*.'s K (Blomberg *et al*., 2003) and Pagel's Lambda (Pagel, 1999) using the ‘phylosig’ function of the R package Phytools (Revell, 2012).

Our empirical analyses were performed at three levels of detail. First, we analysed the allometric relationships between the morphological stomatal traits of all species present in the compiled dataset. Second, we analysed evolutionarily distinct groups of pteridophyte, gymnosperm and angiosperm species in isolation. Third, we analysed selections of amphistomatous monocot and dicot species in isolation. The allometric relationships presented in this study were obtained by fitting a standardized major axis (SMA) regression on log_10_‐transformed values of species average traits using the R package Smatr‐3 (Warton *et al*., 2012). The SMAs fitted on species average traits data included an offset if the offset was significantly different from 0, whereas the SMAs fitted on the PICs were forced through the origin (Garland *et al*., 1992). SMA fits were considered valid when the Pearson product–moment correlation coefficient (*r*) between log_10_‐transformed traits values was significant (*P *<* *0.05).

The first prediction of our theoretical framework (e.g. exponent *S *≤* *−1) was tested based on 95% confidence intervals (CIs) around the SMA regression slope of the allometric relationship between stomatal size and density Eqn 3 To test the second prediction (e.g. Λ < 0) we first derived a mathematical expression for Λ in terms of Eqns 3 and 4 using the Wolfram Mathematica software (see Eqn 5 and Methods S1). The marginal ratio Λ was subsequently calculated from the offsets and exponents found for the allometric relationships as described by Eqns 3 and 4 Uncertainty ranges of Λ were obtained by bootstrapping the (paired) offsets and exponents of the scaling relationships given by Eqns 3 and 4 through resampling the raw data 10 000 times. The prediction that Λ < 0 was subsequently tested based on the 95% CIs of the (bootstrapped) distributions of Λ.

Differences between group means of *g*_smax_, *f*_gc_ and *a*_gc_ were tested with a two‐sided ANOVA. Post‐hoc tests were done with two‐sided Student's *t*‐test assuming unequal sample sizes and variances and included a Bonferroni‐correction considering that three *t*‐tests are performed after each ANOVA. Values of *g*_smax_ were square‐root transformed prior testing, whereas values of *f*_gc_ and *a*_gc_ were log_10_‐transformed.

## Results

### Allometry in stomatal traits

**Figure 3:**
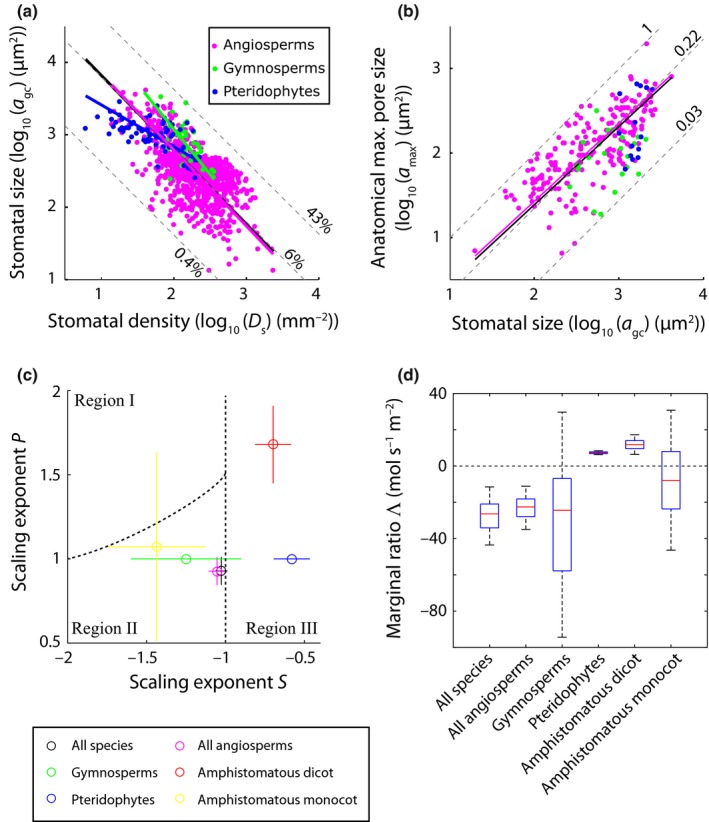
Allometric relationships between morphological stomatal traits. (a) Log_10_‐transformed values of *D*_s_ and *a*_gc_ and (b) *a*_gc_ and *a*_max_. The solid black lines represent standardized major axis (SMA) regressions fitted on all species; the pink, green and blue lines denote the SMAs across subsets of angiosperm, gymnosperm and pteridophyte species, respectively. See Table 1 for detailed statistics. Maximum, median and minimum values of *f*_gc_ (expressed as %), and the ratio *a*_max_ : *a*_gc_ are indicated by the dashed lines in (a) and (b), respectively. (c) Bootstrapped distributions of the scaling exponents *S* and *P* (Eqns 3 and 4, respectively) calculated across the distinct species subsets indicated by the differently coloured symbols. Confidence intervals indicate the 5^th^ and 95^th^ percentiles of the bootstrapped exponents. Dashed lines indicate the borders between the different scaling regions indicated in Fig. 2. (d) Distributions of the bootstrapped values of Λ considering all species present in the compiled dataset and distinct species subsets. The red line inside boxes indicates the median of the bootstrapped distribution, the bottom and top of each box denotes the first and third quartile, respectively, whiskers denote the 5^th^ and 95^th^ percentiles.

We tested our hypothesis and the underlying theoretical framework with observations of stomatal traits drawn from a phylogenetically diverse compilation of published and unpublished data of 1057 species in 156 families from a global range of environments (Tables S1, S3). These data reveal the well‐known inverse relationship between stomatal density *D*_s_ and the size of the guard cell pair *a*_gc_ (*r* ^2^ = 0.43, *P *<* *0.001 for log_10_‐transformed values) (Fig. 3a). The observed exponent *S* in this allometric relationship (e.g. Eqn 3) is negative across all species in our dataset and within subsets of angiosperm, gymnosperm and pteridophyte species (Table 1). Considering this relationship across all species and across the subsets of angiosperm and gymnosperm species provides support for our first prediction that *S* ≤ −1 (see Table 1 for 95% CIs around the exponent *S*), which allows *D*_s_ to increase without increasing *f*_gc_. By contrast, the subset of pteridophyte species shows a shallower inverse relationship with *S *=* *−0.58 with 95% CIs (−0.70, −0.46). The implication is that across the pteridophytes species in our compiled dataset increases in *D*_s_ are associated with increases in *f*_gc_.

**Table 1:** Allometric relationships between morphological stomatal traits

Species selection	X variable	Y variable	Sample size	Offset	Median offset	Lower 95% CI offset	Upper 95% CI offset	Exponent	Median exponent	Lower 95% CI exponent	Upper 95% CI exponent	*r* ^2^	*P*
All species	*D*_s_	*a*_gc_	1032	*b*_s_	0.10	0.04	0.25	*S*	−1.03	−1.07	−0.98	0.43	***
Angiosperms	*D*_s_	*a*_gc_	927	*b*_s_	0.17	0.06	0.47	*S*	−1.05	−1.11	−1.00	0.37	***
Gymnosperms	*D*_s_	*a*_gc_	38	*b*_s_	12	0.02	8.4 × 10^3^	*S*	−1.25	−1.60	−0.90	0.31	***
Pteridophytes	*D*_s_	*a*_gc_	67	*b*_s_	3.0 × 10^−5^	3.9 × 10^−6^	2.3 × 10^−4^	*S*	−0.58	−0.70	−0.46	0.36	***
Amphistomatous dicot	*D*_s_	*a*_gc_	72	*b*_s_	2.1 × 10^−4^	2.4 × 10^−5^	1.7 × 10^−3^	*S*	−0.70	−0.81	−0.58	0.53	***
Amphistomatous monocot	*D*_s_	*a*_gc_	40	*b*_s_	67.00	0.21	2.1 × 10^4^	*S*	−1.43	−1.75	−1.13	0.56	***
All species	*a*_gc_	*a*_max_	251	*b*_p_	0.05	0.01	0.29	*P*	0.92	0.84	1.01	0.50	***
Angiosperms	*a*_gc_	*a*_max_	214	*b*_p_	0.05	0.01	0.31	*P*	0.92	0.84	1.00	0.58	***
Gymnosperms	*a*_gc_	*a*_max_	23	*b*_p_	–	–	–	*P*	–	–	–	–	ns
Pteridophytes	*a*_gc_	*a*_max_	14	*b*_p_	–	–	–	*P*	–	–	–	–	ns
Amphistomatous dicot	*a*_gc_	*a*_max_	19	*b*_p_	5.0 × 10^5^	3.8 × 10^3^	6.5 × 10^7^	*P*	1.68	1.45	1.91	0.93	***
Amphistomatous monocot	*a*_gc_	*a*_max_	10	*b*_p_	1.17	9.7 × 10^−6^	1.5 × 10^5^	*P*	1.07	0.51	1.63	0.59	**

Offsets and exponents reflect standardized major axis regressions calculated on the species average trait data expressed in the following units: stomatal density (*D*_s_, no. stomata∙m^−2^), average size of the guard cell pair (*a*_gc_, m^2^), anatomical maximum aperture (*a*_max_, m^2^). The *r* ^2^ denotes the Pearson product–moment correlation coefficient between log_10_‐transformed traits values. Significance levels of correlation are indicated: ***, *P *<* *0.001; **, *P *<* *0.01; ns, *P *≥* *0.05.

Our results further show an allometric relationship between the size of the guard cell pair *a*_gc_ and the anatomical maximum pore size *a*_max_ across all species in the dataset (*P *<* *0.001, *r* ^2^ = 0.50 for log_10_‐transformed values), and within the subset of angiosperm species (*P *<* *0.001, *r* ^2^ = 0.58 for log_10_‐transformed values) (Fig. 3b). The observed exponent *P* in these relationships (e.g. Eqn 4) cannot be distinguished from shape‐preserving unity (see Table 1 for 95% CIs around the exponent *P*). No significant relationship (correlation *P *>* *0.05 for log_10_‐transformed values) was found between these traits considering the subsets of gymnosperm and pteridophyte species in isolation.

In order to control for potential phylogenetic bias in our data we also obtained the allometric relationships between Phylogenetically Independent Contrasts (PICs) (Felsenstein, 1985) of the traits considered here (Fig. S3). This analysis suggests that the observed relationships are robust to phylogenetic bias (Table S4) despite all traits reflecting phylogenetic signal (Table S5).

### Optimal allocation of epidermal area

In order to test whether the combination of observed allometric relationships given by Eqns 3 and 4 supports our second prediction that Λ < 0 and fall in the region (II) indicated in Fig. 2, we obtained bootstrapped distributions of these relationships considering all species in the compiled dataset and considering subsets of angiosperm, gymnosperm and pteridophyte species in isolation. Combinations of the observed exponents *S* and *P* and the resulting consequence for the trade‐off between changes in *g*_smax_ and *f*_gc_ are shown in Fig. 3(c). In support of our hypothesis, we observed that Λ < 0 across all species and across the subset of angiosperm species (Fig. 3d). As we found no significant scaling relationship between the size of the guard cell pair *a*_gc_ and the anatomical maximum pore size *a*_max_ across the subsets of gymnosperm and pteridophyte species, we principally rejected our hypothesis considering these clades in isolation. However, the lack of a significant relationship between *a*_max_ and *a*_gc_ in the gymnosperm and pteridophyte clades could be related to the relatively small sample size (Table 1). Hence, we calculated Λ for the subsets of gymnosperm and pteridophyte species assuming that the generic scaling relationship between *a*_max_ and *a*_gc_ applies to these clades in isolation. For the subset of pteridophyte species we then observed a marginal ratio Λ > 0, whereas for gymnosperms the median value of Λ was negative but, considering a 95% confidence limit, this result is statistically not significant (Fig. 3d).

**Figure 4:**
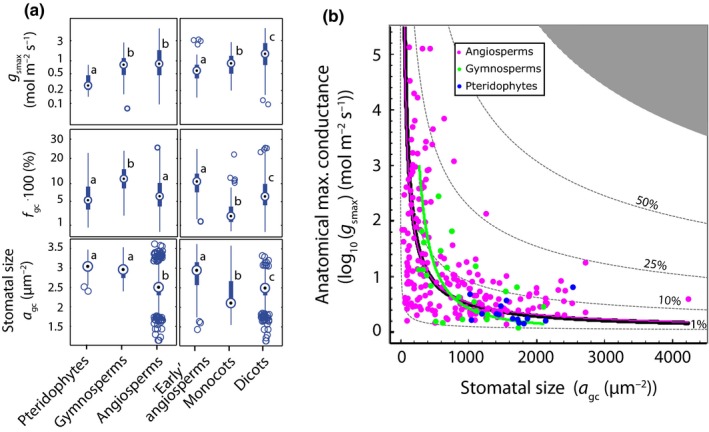
Reduction of stomatal size extends viable ranges in *g*_smax_. (a) Box plots of *g*_smax_ (upper panels), *f*_gc_ (middle panels) and *a*_gc_ (lower panels) separated for pteridophytes, gymnosperms and angiosperms (left) and three angiosperm subclades (right). The early angiosperm clade includes the ANITA‐grade, Chloranthales and magnoliids. Circles in boxes indicate the median, the bottom and top of each box denotes the first and third quartile, respectively, and whiskers denote the 5^th^ and 95^th^ percentiles. Data points outside whiskers are shown individually. Letters denote significant differences between group means (*P *<* *0.05, with Bonferroni correction). (b) Two‐dimensional morphospace expressed in terms of *a*_gc_ and *g*_smax_. Data points indicate species‐averaged combinations *a*_gc_ and *g*_smax_, with *g*_smax_ calculated following Eqn 1 The solid black line represent the standardized major axis (SMA) regressions fitted on all species, the pink and green lines denote the SMAs across subsets of angiosperm and gymnosperm species, respectively. See Table 1 and Supporting Information Table S4 for detailed statistics. Dashed lines indicate *f*_gc_ as a percentage.

The proposed theoretical framework implies that spatially optimal stomatal scaling requires coordinated evolution of stomatal densities and stomatal morphology in relation to evolution towards higher *g*_smax_. This inference is supported by increases in *g*_smax_ between consecutive evolutionary groups that are not associated with changes in *f*_gc_ owing to concurrent reductions in the size of the guard cell pair *a*_gc_ (Fig. 4a). Further support for this inference is found in the inverse relationship between species averages of *g*_smax_ and stomatal size considering all species together, and within the subsets of angiosperms and gymnosperms (Figs 4b, S3c). We note that these results do not imply that species with small stomata necessarily have high *g*_smax_. Rather, smaller stomata extend the upper range of *g*_smax_ that can viably be obtained within the constraints set by *f*_gc_ without necessarily limiting the potential to develop leaves with low *g*_smax_.

### Amphistomaty

Amphistomatous leaves provide an example were the proposed spatial constraint on stomatal size‐density combinations may be less pressing owing to the doubling of available epidermal space compared with hypostomatous leaves. Relaxing the spatial constraint on stomatal size‐density combinations theoretically allows for the slope *S* in the allometric relationship between *D*_s_ and *a*_gc_ to be shallower than the area‐preserving value of –1. It may therefore be expected that stomatal scaling relationships for amphistomatous angiosperm species result in Λ > 0 and fall in the region (III) of Fig. 2.

As the subset of all angiosperm species reflects the proposed optimal scaling of stomatal traits, we examined whether a subset of amphistomatous angiosperms deviates from this spatially optimal pattern. The amphistomatous angiosperm species selection consists of dicots and monocots (mostly grasses) which are phylogenetically distinct clades and have distinct leaf morphologies. We therefore analysed the allometric relationships for these groups separately. In line with a relaxation of the spatial constraint, the subgroup of amphistomatous dicots shows allometric relationships with Λ > 0 (Fig. 3c,d) that fall in the region (III) of Fig. 2, specifically because the exponent *S *>* *−1 (Fig. S4). Hence, increases in *g*_smax_ are tied to increases in *f*_gc_ across this group of species. By contrast, we found that *S *<* *−1 for the subset of amphistomatous monocots. As a result, the median of the bootstrapped allometric relationships for amphistomatous monocots results in Λ < 0 and falls in region (II) of Fig. 2, with the 95% CIs overlapping with region (I), owing to the relatively large spread in the observed exponent *P*.

## Discussion

Our theoretical framework and empirical analyses suggest that the evolution of morphological stomatal traits involves a trade‐off to maximize the gas exchange capacity of the epidermis while minimizing the fraction of the epidermis that is covered by stomata. This result is in line with fossil evidence (Franks & Beerling, 2009) which suggests that evolutionary increases in *g*_smax_ were closely related to increases in stomatal densities and decreases in stomatal (pore) size (Assouline & Or, 2013), especially by evolution within the angiosperm clade (de Boer *et al*., 2012). These stomatal size–density relationships may have evolved because variations in stomatal density are controlled by cell‐to‐cell signalling mechanisms that regulate spacing between stomata (Lee *et al*., 2015) as well as phenotypic adjustments to environmental conditions (Bergmann & Sack, 2007; Doheny‐Adams *et al*., 2012). Stomatal size is less plastic than stomatal density (Zhang *et al*., 2012) owing to its relationship with genome size (Beaulieu *et al*., 2008; Franks *et al*., 2012a). Still, the association between genome size and stomatal size may change slightly under environmental pressure (Lomax *et al*., 2009; Jordan *et al*., 2015) and thereby facilitate adaptive evolution of stomatal (pore) size in coordination with changes in stomatal density. Smaller stomata may also allow for faster dynamic responses in stomatal aperture (Drake *et al*., 2013), a benefit that may specifically confer gas exchange advantage to angiosperms owing to their accurate control on stomatal aperture (Brodribb *et al*., 2009; McAdam & Brodribb, 2012, 2014).

Our results provide no support for spatially optimal co‐evolution of stomatal traits occurring within the pteridophyte and gymnosperm clades. The lack of clear stomatal scaling relationships within these clades could be related to the relatively small sample size in relation to their relatively narrow ranges in stomatal size–density combinations. In contrast to angiosperms that occupy a wide morphospace in terms of stomatal size–density combinations, pteridophytes and gymnosperms are restricted to combinations of relatively few and large stomata. These narrow morphological ranges could reflect limited selection pressure on stomatal morphology in relation to *g*_smax_. In contrast to angiosperms, the leaf gas exchange capacity of gymnosperm and pteridophyte leaves is restricted by their relatively low leaf water transport capacity (Brodribb *et al*., 2005). Owing to the close relationships between leaf water transport capacity and stomatal gas exchange (Sack & Scoffoni, 2013) species from these clades may experience little competitive advantage from increasing *g*_smax_ by adjusting stomatal morphology because the leaf water transport capacity is not sufficient to keep the stomata open under typical growth conditions (McElwain *et al*., 2015).

Yet, the success of species with relatively large stomata suggests that this morphology, despite incurring potential functional limitations, does not threaten their survival. However, it might be reflective of adaptation to specific environmental niches. The angiosperm family Liliaceae, for example, contains a relatively large proportion of species with large stomata but these are restricted predominantly to regions with low spring temperatures (Leitch *et al*., 2007). Similarly, the persistence of large stomata in the pteridophyte clade hints at limited selection for high *g*_smax_ owing to their relatively low leaf water transport capacity and predominant occurrence in low‐light environments (Brodribb *et al*., 2005, 2007). The large stomata in the gymnosperm clade could be associated with their relatively low leaf water transport capacity (Brodribb *et al*., 2005, 2007) in relation to environments that select for leaf longevity rather than high productivity (Bond, 1989; Reich *et al*., 2014). A similar mechanism could be invoked to explain the difference in stomatal scaling relationships between the subgroups of amphistomatous dicots and monocots. Monocots have, on average, sturdier leaves with longer life spans (Onoda *et al*., 2011) and distinctly lower leaf vein densities than other angiosperm subclades (Roddy *et al*., 2013). As a result, monocots may experience little evolution pressure to increase leaf gas exchange capacity despite having both leaf sides available to allocate to stomata. Hence, the competitive advantage of spatially optimal allocation of leaf epidermal area to stomata could be negated by specific growth conditions in relation to leaf hydraulics and leaf morphology.

Our results highlight that the stomatal morphology of angiosperms evolved towards higher *g*_smax_ along spatially optimal allometric relationships. As a result, this clade now occupies a specific part of the morphospace that is associated with smallest stomata. Angiosperms thereby extend the upper range of *g*_smax_ beyond those of gymnosperms and pteridophytes without limiting the possibility to develop leaves with low *g*_smax_ by downregulating stomatal density. The resulting wide range of viable *g*_s_–*g*_smax_ combinations equips angiosperms with developmental and evolutionary flexibility in leaf gas exchange (McElwain *et al*., 2015) that, in combination with innovations of leaf water transport tissue (Feild *et al*., 2011; de Boer *et al*., 2012), enables them to thrive in diverse and ever‐changing global climates.

## Author contributions

H.J.B., C.A.P. and E.J.V. designed the research. H.J.B., S.C.D., P.J.F. and C.A.P. analysed data, H.J.B., P.J.F., E.J.V. and F.W‐C. contributed data. H.J.B. wrote the manuscript. All authors discussed the results and commented on the manuscript.

## Supporting information

Please note: Wiley Blackwell are not responsible for the content or functionality of any supporting information supplied by the authors. Any queries (other than missing material) should be directed to the *New Phytologist* Central Office.

**Fig. S1** Relationship between guard cell length and width.
**Fig. S3** Allometry between independent contrasts.
**Fig. S4** Allometric relationships between stomatal traits of amphistomatous monocots and dicots.
**Table S1** References to original data sources used in the compiled dataset on species average stomatal traits
**Table S2** Geometric constant *f*_lw_ used for calculating *g*_smax_ for different stomata types
**Table S4** Allometric relationships between phylogenetically independent contrasts of the morphological stomatal traits
**Table S5** Test for phylogenetic signal in the traits considered based on Blomberg *et al*.'s K and Pagel's λ
**Methods S1** Detailed derivation and expression of the marginal ratio Λ.

**Fig. S2** Phylogenetic tree of all species included in the stomatal trait dataset.

**Table S3** Compilation of species average stomatal trait values

**Notes S1** Script file to be opened with Wolfram Mathematica software containing the derivation and expression of the marginal ratio Λ.
