# Supplementary material for: Optimal allocation of leaf epidermal area for gas exchange
Source: New Phytol. 2016 Mar 16;210(4):1219–28. doi: 10.1111/nph.13929 (PMC5069575; doi:10.1111/nph.13929)
Supplement: Supplementary file 1 — Fig. S1 Relationship between guard cell length and width. Fig. S3 Allometry between independent contrasts. Fig. S4 Allometric relationships between stomatal traits of amphistomatous monocots and dicots. Table S1 References to original data sources used in the compiled dataset on species average stomatal traits Table S2 Geometric constant f lw used for calculating g smax for different stomata types Table S4 Allometric relationships between phylogenetically independent contrasts of the morphological stomatal traits Table S5 Test for phylogenetic signal in the traits considered based on Blomberg et al.'s K and Pagel's λ Methods S1 Detailed derivation and expression of the marginal ratio Λ. [file NPH-210-1219-s001.pdf]

New Phytologist

***New Phytologist* Supporting Information Figs S1, S3 & S4, Tables S1, S2, S4 & S5 and Methods S1**

Title: Optimal allocation of leaf epidermal area for gas exchange

Authors: Hugo J. de Boer, Charles A. Price, Friederike Wagner-Cremer, Stefan C. Dekker, Peter J. Franks and Erik J. Veneklaas

Article acceptance date: 8 February 2016

The following Supporting Information is available for this article:

Fig. S1 Relationship between guard cell length and width.

Fig. S2 Phylogenetic tree of the species included in the stomatal trait data set (separate PDF file).

Fig. S3 Allometry between independent contrasts.

Fig. S4 Allometric relationships between stomatal traits of amphistomatous monocots and dicots.

Table S1 References to original data sources used in the compiled data set on stomatal traits

Table S2 Geometric constant f_{lw} used for calculating g_{smax} for different stomata types

Table S3 Compilation of species average stomatal trait values (separate Excel file)

Table S4 Allometric relationships between phylogenetically independent contrasts of the morphological stomatal traits

Table S5 Test for phylogenetic signal in the traits considered based on Blomberg *et al*.'s K and Pagel's λ

Methods S1 Detailed derivation and expression of the marginal ratio Λ.

Notes S1 Script file to be opened with Wolfram Mathematica software (developed with version 8.0.1.0) containing the derivation and expression of the marginal ratio Λ (separate file).

Methods S1 Detailed derivation and expression of the marginal ratio Λ.

Derivation of the marginal ratio Λ

To obtain the marginal ratio Λ we expressed the change in g_{smax} due to a change in D_s and associated changes in stomatal morphology ($\partial g_{s\max} / \partial D_S$), relative to the resulting change in fractional stomatal cover ($\partial f_{gc} / \partial D_S$), as shown in Eqn 5 in the main text. Hereto we expressed f_{gc} and g_{smax} (Eqns 1 and 2, respectively) in terms of the allometric scaling relationships given by Eqns 3 and 4. This expression for the marginal ratio Λ is obtained as follows.

Substituting Eqn 4 in Eqn 2 yields an expression for g_{smax} in terms of D_s and a_{gc}:

$$g_{s\max} = \frac{D_s b_p {a_{gc}}^P 2 \frac{d_{H2O}}{w_v}}{\sqrt{b_p {a_{gc}}^P} \sqrt{\pi} + 2\sqrt{\frac{2}{\pi}} \sqrt{a_{gc} r_{dl}}} \quad \text{Eqn 8}$$

Substitution of Eqn 3 in the above result yields:

$$g_{s\max} = \frac{D_s b_p (b_s {D_s}^S)^P 2 \frac{d_{H2O}}{w_v}}{\sqrt{b_p (b_s {D_s}^S)^P} \sqrt{\pi} + 2\sqrt{\frac{2}{\pi}} \sqrt{(b_s {D_s}^S) r_{dl}}} \quad \text{Eqn 9}$$

Derivation with respect do D_s yields the (marginal) change in g_{smax} due to a change in D_s:

$$\frac{\partial g_{s\max}}{\partial D_s} = \frac{b_p \cdot \left(b_s {D_s}^S\right)^P \cdot \left(\sqrt{b_p (b_s {D_s}^S)^P} \pi \sqrt{b_S {D_s}^S r_{dl}} \left(2 + P \cdot S\right) + 2\sqrt{2} \cdot b_S {D_S}^S r_{dl} \left(2 + \left(2P - 1\right)S\right)\right) \cdot \frac{d_{H2O}}{w_v}}{\sqrt{\pi} \sqrt{b_S {D_S}^S r_{dl}} \left(\sqrt{b_P (b_S {D_S}^S)^P} \sqrt{\pi} + 2\sqrt{\frac{\pi}{2}} \sqrt{b_S {D_S}^S r_{dl}}\right)^2} \quad \text{Eqn 10}$$

Substitution of Eqn 3 in Eqn 1 yields an expression for f_{gc} in terms of D_s:

$$f_{gc} = b_s {D_s}^{1+S} . \quad \text{Eqn 11}$$

Derivation with respect do D_s yields the marginal change in fractional stomatal cover due to a change in D_s:

$$\frac{\partial f_{gc}}{\partial D_s} = b_s {D_s}^S (1+S) \qquad \text{Eqn 12}$$

Hence, the marginal ratio Λ, the ratio of Eqn 10 to Eqn 12, is expressed as:

$$\Lambda = \frac{\partial g_{s\max} / \partial D_s}{\partial f_{gc} / \partial D_s} = \frac{\dfrac{b_p \cdot \left(b_s {D_s}^S\right)^P \cdot \left(\sqrt{b_p (b_s {D_s}^S)^P}\, \pi \sqrt{b_S {D_s}^S r_{dl}} \left(2 + P \cdot S\right) + 2\sqrt{2} \cdot b_S {D_S}^S r_{dl} \left(2 + \left(2P-1\right)S\right)\right) \cdot \frac{d_{H2O}}{w_v}}{\sqrt{\pi}\sqrt{b_S {D_S}^S r_{dl}} \left(\sqrt{b_P (b_S {D_S}^S)^P}\, \sqrt{\pi} + 2\sqrt{\frac{\pi}{2}} \sqrt{b_S {D_S}^S r_{dl}} \right)^2}}{b_s {D_s}^S (1+S)} \qquad \text{Eqn 13}$$

A script file for Wolfram Mathematica containing this derivation of Λ is provided separately with these Supporting Information Methods (Notes S1).

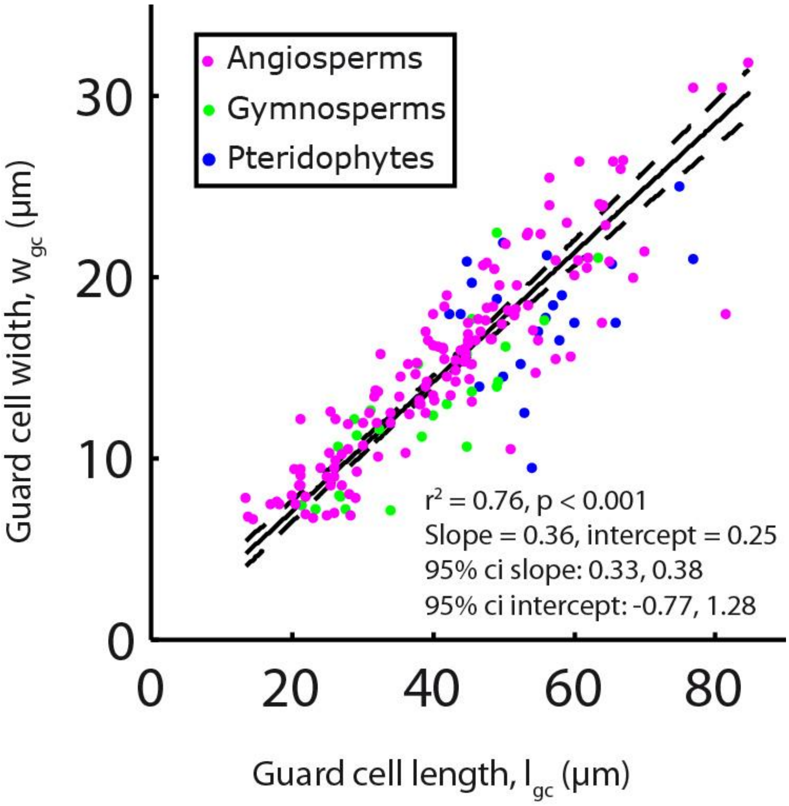

Fig. S1 Relationship between guard cell length (l_{gc}) and width (l_{gc}). Statistics on the standard major axis (SMA) regression fitted across all species in the data set are indicated in the figure. The intercept of the SMA could not be distinguished from 0 and was therefore fitted through the origin. The parameter $r_{wl} = w_{gc}/l_{gc}$ is estimated at 0.36 based on the SMA slope.

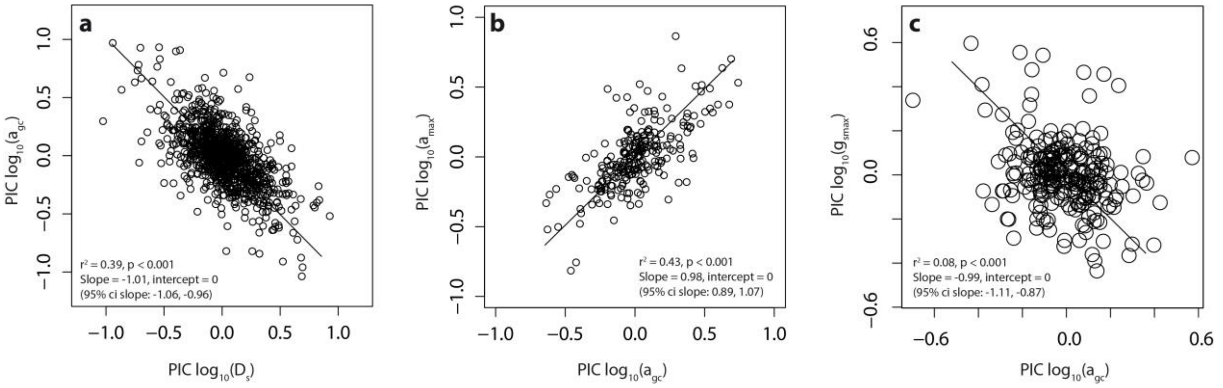

Fig. S3 Allometry between independent contrasts. SMA regressions between the phylogenetic independent contrasts (PICs) (Felsenstein, 1985) of $\log_{10}$-transformed values of (a) D_s and a_{gc}, (b) a_{gc} and a_{max} and (c) a_{gc} and g_{smax}. SMA regressions were fitted through the origin (Garland *et al.*, 1992). Statistics on the SMA regressions are plotted in the panels, with further details on statisticsprovided in Table S4.

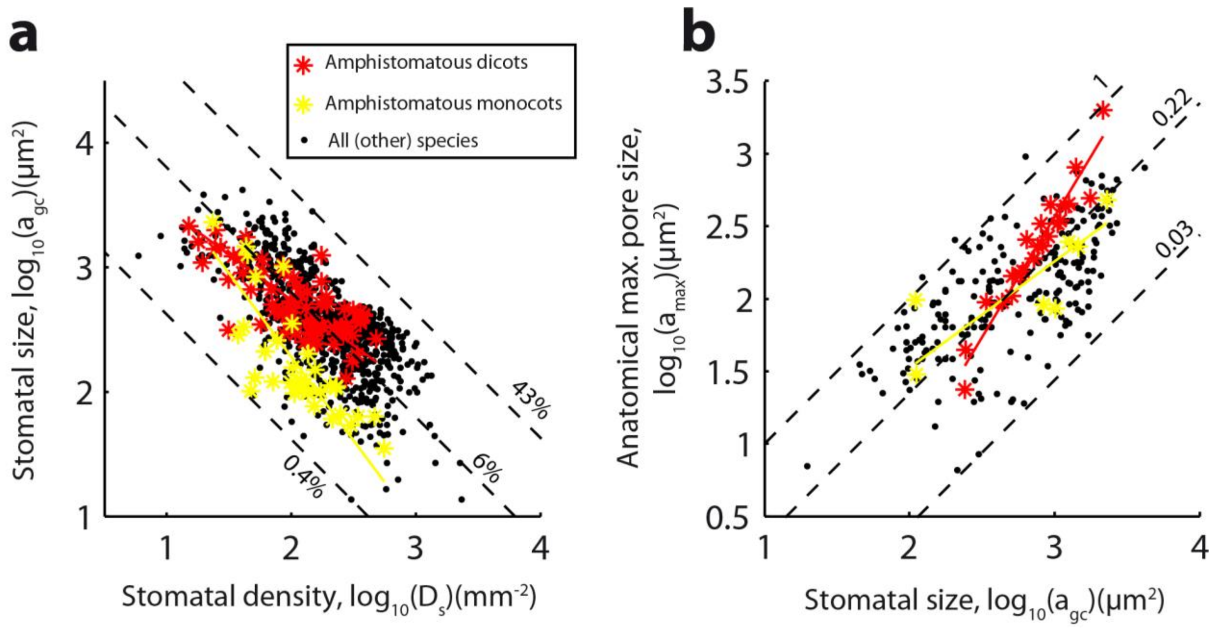

Fig. S4 Allometric relationships between stomatal traits of amphistomatous monocots and dicots. (a) Log_{10}-transformed values of D_s and a_{gc} and (b) a_{gc} and a_{max}. The solid lines represent SMA regressions fitted on amphistomatous monocots (yellow) and dicots (red). Maximum, median and minimum values of f_{gc} (expressed as %), and the ratio $a_{max} : a_{gc}$ are indicated by the dashed lines in (a) and (b), respectively. Detailed statistics on the SMA regressions are provided in Table S4.

Table S1 References to original data sources used in the compiled data set on species average stomatal traits (see Table S3)

Author	Experimental facility	Location of natural site or garden
Abrams (1987)	Field	Northeast Kansas, USA
Abrams & Kubiske (1990)	Field	Central Wisconsin, USA
Anoruo & Blake (1997)	Field	South Eastern USA
Batos *et al.* (2010)	Field	Northern Serbia
H. J. de Boer unpublished a	Field	Western Australia
H. J. de Boer unpublished b	Garden	Botanical garden Utrecht, the
Bongers & Popma (1990)	Field	Netherlands
Brodribb *et al.* (2013)	Field	Los Tuxtlas, Veracruz, Mexico
Bruschi *et al.* (2000)	Field	Diverse, southern hemisphere
Brutti *et al.* (2002)	Glasshouse	Northern and central Italy
Camargo & Marenco (2011)	Field	-
Carpenter & Smith (1975)	Field	Central Amazonia, Brazil
Chiba & Watanabe (1952)	Garden	Diverse
Corneanu *et al.* (2004)	Garden	Japan
Cornelissen *et al.* (2003)	Field & Growth	Romania
Eckerson (1908)	chamber	Central England and Northern Spain
Fahmy (1997)	Field	Diverse
Feild *et al.* (2011b)	Field	Egypt
F. Pérez unpublished	Field	Diverse, tropical
P. J. Franks unpublished a	Field	Central Chile
P. J. Franks unpublished b	Garden	Royal Botanic Gardens, Sydney,
Franks *et al.* (2009)	Growth chamber	Australia
Gibson (1983)	Field	-
Gindel (1969)	Field	South-western Australia
Haworth *et al.* (2011)	Field	Semi-arid and arid North America
Hietz & Briones (1998)	Growth chamber	Diverse, Israel

Holland & Richardson (2009)	Field	-
Kawamitsu *et al.* (1996)	Field	Central Veracruz, Mexico
Lammertsma *et al.* (2011)	Garden	White Mountains, New Hampshire,
Lavalle *et al.* (2007)	Field	USA
Locosselli & Ceccantini (2012)	Field	Japan
MacDaniels & Cowart (1944)	Field	Florida, USA
Meidner & Mansfield (1968)	Field	Diverse, South America
Mitton *et al.* (1998)	Field & Glasshouse	Brazil
Nóbrega & Pereira (1992)	Field	United Kingdom
Pallardy & Kozlowski (1979)	Field	United Kingdom
Pyakurel & Wang (2014)	Glasshouse	Colorado, USA
Qing-Wen *et al.* (2005)	Glasshouse	Besteiros, Portugal
Richardson *et al.* (2001)	Garden	-
Rolleri *et al.* (2012)	Field	-
Roth (1984)	Field	China and USA
Russo *et al.* (2010)	Field	Northwestern British Columbia,
Rutter & Willmer (1979)	Field	Canada
Sha Valli Khan *et al.* (1999)	Glasshouse	Diverse, South America
Stenström *et al.* (2002)	Glasshouse	Venezuela
Tanner & Kapos (1982)	Garden	Lambir Hills National Park, Sarawak,
Taylor *et al.* (2012)	Field	Malaysia
Tiwari *et al.* (2013)	Glasshouse	-
Toral *et al.* (2010)	Field & Garden	-
Vygodskaya *et al.* (1997)	Field	Tromsø, Norway
Wagner *et al.* (1996)	Field	Blue Mountains, Jamaica
Wagner *et al.* (2000)	Field	-
F. Wagner-Cremer	Field	Kumaun Mountains, India
unpublished a	Field	Diverse, Chile
F. Wagner-Cremer	Field	Siberia, Russia
unpublished b	Field	Mariapeel, the Netherlands
Wang *et al.* (2014)	Field	Kevo, Utsjoki, Finnish Lapland

Zhang *et al.* (2012)<br>Zhang *et al.* (2014)	Garden	Netherlands<br>Florida<br>Changbai Mountain, China<br>Qilian Mountains, China<br>Xishuangbanna Botanical Garden, China

Table S2 Geometric constant f_{lw} used for calculating g_{smax} for different stomata types

**Stomata type**	**Maximum pore area / area of circle with diameter = *p*** $f_{lw} = a_{max}/(\pi \cdot l_p^2/4)$
Fern and gymnosperm type	0.5
Ginkgo type	0.6
Angiosperm type- small (<30 µm length)	1
Angiosperm type- large (>30 µm length)	1
Angiosperm grass type- small (<30 µm length)	0.5
Angiosperm grass type- large (>30 µm length)	0.4

l_p, stomatal pore length; a_{max}, maximum area of stomatal pore for maximally open stoma; π, mathematical constant. Values are based on Franks *et al*. (2014).

New Phytologist

Table S4 Allometric relationships between phylogenetic independent contrasts (PICs) of species average stomatal trait values

Species selection	X variable	Y variable	df	Intercept	Median slope	Lower 95% CI slope	Upper 95% CI slope	r^2	*P*
All species	PIC of D_s	PIC of a_{gc}	1023	0	-1.01	-1.06	-0.96	0.39	***
Angiosperms	PIC of D_s	PIC of a_{gc}	921	0	-1.00	-1.06	-0.95	0.32	***
Gymnosperms	PIC of D_s	PIC of a_{gc}	35	0	-1.36	-1.78	-1.03	0.33	***
Pteridophytes	PIC of D_s	PIC of a_{gc}	61	0	-0.67	-0.83	-0.54	0.29	***
All species	PIC of a_{gc}	PIC of a_{max}	248	0	0.98	0.89	1.07	0.43	***
Angiosperms	PIC of a_{gc}	PIC of a_{max}	211	0	1.02	0.93	1.12	0.51	***
Gymnosperms	PIC of a_{gc}	PIC of a_{max}	20	0	1.24	0.83	1.85	0.17	*
Pteridophytes	PIC of a_{gc}	PIC of a_{max}	13	0	-	-	-	-	ns
All species	PIC of a_{gc}	PIC of g_{smax}	248	0	-0.99	-1.11	-0.87	0.01	***

Intercepts and slopes reflect SMA regressions calculated across the PICs of the traits considered with the SMA regressions forced through the origin. The r^2 denotes the Pearson product-moment correlation coefficient between PICs. Significance levels of this correlation are indicated: ***, $P < 0.001$; *, $P < 0.05$; ns, $P \geq 0.05$.

Table S5 Test for phylogenetic signal in the traits considered based on Blomberg *et al*.'s K (Blomberg *et al.*, 2003) and Pagel's λ (Pagel, 1999)

Trait	Blomberg *et al*.'s K	Pagel's λ
$Log_{10}(D_s)$	0.34***	0.93***
$Log_{10}(a_{gc})$	0.45***	0.89***
$Log_{10}(a_{max})$	0.14***	0.88***
$Log_{10}(g_{smax})$	0.39***	0.93***
$Log_{10}(f_{gc})$	0.35***	0.91***

Significant evolutionary signal is indicated: ***, $P \leq 0.001$.

References

Abrams MD. **1987**. Leaf structural and photosynthetic pigment characteristics of three gallery-forest hardwood species in northeast Kansas. *Forest Ecology and Management* **22**: 261–266.

Abrams MD, Kubiske ME. **1990**. Leaf structural characteristics of 31 hardwood and conifer tree species in central Wisconsin: Influence of light regime and shade-tolerance rank. *Forest Ecology and Management* **31**: 245–253.

Anoruo AO, Blake JI. **1997**. Variation in guard cell size, interstomatal spacing and stomatal frequency in longleaf pine along latitudinal and longitudinal gradients. *Journal of Sustainable Forestry* **5**: 169–178.

Batos B, Vilotic D, Orlovic S, Miljkovic D. **2010**. Inter and intra-population variation of leaf stomatal traits of *Quercus robur* L. in Northern Serbia. *Archives of Biological Sciences* **62**: 1125–1136.

Blomberg SP, Garland T, Ives AR. **2003**. Testing for phylogenetic signal in comparative data: behavioral traits are more labile. *Evolution* **57**: 717–745.

Bongers F, Popma J. **1990**. Leaf characteristics of the tropical rain forest flora of Los Tuxtlas, Mexico. *Botanical Gazette* **151**: 354–365.

Brodribb TJ, Jordan GJ, Carpenter RJ. **2013**. Unified changes in cell size permit coordinated leaf evolution. *New Phytologist* **199**: 559–570.

Bruschi P, Vendramin GG, Bussotti F, Grossoni P. **2000**. Morphological and molecular differentiation between *Quercus petraea* (Matt.) Liebl. and *Quercus pubescens* Willd. (Fagaceae) in Northern and Central Italy. *Annals of Botany* **85**: 325–333.

Brutti CB, Rubio EJ, Llorente BE, Apóstolo NM. **2002**. Artichoke leaf morphology and surface features in different micropropagation stages. *Biologia Plantarum* **45**: 197–204.

Camargo MAB, Marenco RA. **2011**. Density, size and distribution of stomata in 35 rainforest tree species in Central Amazonia. *Acta Amazonica* **41**: 205–212.

Carpenter SB, Smith ND. **1975**. Stomatal distribution and size in southern Appalachian hardwoods. *Canadian Journal of Botany* **53**: 1153–1156.

Chiba S, Watanabe M. **1952**. Tetraploids of *Larix Kaempferi* appeared in the nurseries. *Journal of the Japanese Forestry Society* **34**: 276–278.

Corneanu G, Corneanu M, Bercu R. **2004**. Comparison of some morpho-anatomical features at fossil vegetal species and their actual correspondent species. *Studia UBB Geologia* **49**: 77–84.

Cornelissen JHC, Cerabolini B, Castro-Díez P, Villar-Salvador P, Montserrat-Martí G, Puyravaud JP, Maestro M, Werger MJA, Aerts R. **2003**. Functional traits of woody plants: correspondence of species rankings between field adults and laboratory-grown seedlings? *Journal of Vegetation Science* **14**: 311–322.

Eckerson S. **1908**. The number and size of the stomata. *Botanical Gazette* **46**: 221–224.

Fahmy GM. **1997**. Leaf anatomy and its relation to the ecophysiology of some non-succulent desert plants from Egypt. *Journal of Arid Environments* **36**: 499–526.

Feild TS, Upchurch Jr GR, Chatelet DS, Brodribb TJ, Grubbs KC, Samain M-S, Wanke S. **2011**. Fossil evidence for low gas exchange capacities for Early Cretaceous angiosperm leaves. **37**: 195–213.

Felsenstein J. **1985**. Phylogenies and the comparative method. *The American Naturalist* **125**: 1–15.

Franks PJ, Drake PL, Beerling DJ. **2009**. Plasticity in maximum stomatal conductance constrained by negative correlation between stomatal size and density: an analysis using *Eucalyptus globulus*. *Plant, Cell & Environment* **32**: 1737–1748.

Franks PJ, Royer DL, Beerling DJ, Van de Water PK, Cantrill DJ, Barbour MM, Berry JA. **2014**. New constraints on atmospheric CO_2 concentration for the Phanerozoic. *Geophysical Research Letters* **41**: 2014GL060457.

Garland T, Harvey PH, Ives AR. **1992**. Procedures for the Analysis of comparative data using phylogenetically independent contrasts. *Systematic Biology* **41**: 18–32.

Gibson AC. **1983**. Anatomy of Photosynthetic Old Stems of Nonsucculent Dicotyledons from North American Deserts. *Botanical Gazette* **144**: 347–362.

Gindel I. **1969**. Stomatal number and size as related to soil moisture in tree xerophytes in Israel. *Ecology* **50**: 263–267.

Haworth M, Fitzgerald A, McElwain JC. **2011**. Cycads show no stomatal-density and index response to elevated carbon dioxide and subambient oxygen. *Australian Journal of Botany* **59**: 630–639.

Hietz P, Briones O. **1998**. Correlation between water relations and within-canopy distribution of epiphytic ferns in a Mexican cloud forest. *Oecologia* **114**: 305–316.

Holland N, Richardson AD. **2009**. Stomatal length correlates with elevation of growth in four temperate species. *Journal of Sustainable Forestry* **28**: 63–73.

Kawamitsu Y, Agata W, Hiyane S, Murayama S, Nose A, Shinjyo C. **1996**. Relation between leaf gas exchange rate and stomata, 1: stomatal frequency and guard cell length in C_3 and C_4 grass species. *Japanese Journal of Crop Science* **65**: 626–633.

Lammertsma EI, Boer HJ de, Dekker SC, Dilcher DL, Lotter AF, Wagner-Cremer F. **2011**. Global CO_2 rise leads to reduced maximum stomatal conductance in Florida vegetation. *Proceedings of the National Academy of Sciences, USA* **108**: 4035–4040.

Lavalle MDC, Gardella MC, Cortizo L, Bodnar J, Rodríguez M. **2007**. Implicación taxonómica de estudios morfológicos comparativos en *Blechnum* L. (Blechnaceae – Pteridophyta). *Botanica Complutensis* **31**: 75 – 85.

Locosselli GM, Ceccantini G. **2012**. Plasticity of stomatal distribution pattern and stem tracheid dimensions in *Podocarpus lambertii*: an ecological study. *Annals of Botany* **110**: 1057–1066.

MacDaniels LH, Cowart FF. **1944**. The development and structure of the apple leaf. *Memoir Cornell University Agricultural Experiment Station* **258**: 1–29.

Meidner H, Mansfield TA. **1968**. *Physiology of Stomata*. London, UK: McGraw-Hill.

Mitton JB, Grant MC, Yoshino AM. **1998**. Variation in allozymes and stomatal size in pinyon (*Pinus edulis*, Pinaceae), associated with soil moisture. *American Journal of Botany* **85**: 1262–1265.

Nóbrega CM, Pereira JS. **1992**. Gradients of anatomy and morphology of leaves in the crowns of cork oak. *Scientia gerundensis* **18**: 53–60.

Pagel M. **1999**. Inferring the historical patterns of biological evolution. *Nature* **401**: 877–884.

Pallardy SG, Kozlowski TT. **1979**. Frequency and length of stomata of 21 *Populus* clones. *Canadian Journal of Botany* **57**: 2519–2523.

Pyakurel A, Wang JR. **2014**. Leaf morphological and stomatal variations in paper birch populations along environmental gradients in Canada. *American Journal of Plant Sciences* **05**: 1508–1520.

Qing-Wen M, Feng-Lan L, Cheng-Sen L. **2005**. Leaf epidermal structure and stomatal parameters of the genus *Taxodium* (Taxodiaceae). *Acta Phytotaxonomica Sinica* **43**: 517.

Richardson AD, Ashton PMS, Berlyn GP, McGroddy ME, Cameron IR. **2001**. Within-crown foliar plasticity of western hemlock, *Tsuga heterophylla*, in relation to stand age. *Annals of Botany* **88**: 1007–1015.

Rolleri CH, Prada C, Passarelli L, Galán JMG y, Ciciarelli M de las M. **2012**. Revisión de especies monomórficas y subdimórficas del género 'Blechnum' ('Blechnaceae-Polypodiophyta'). *Botanica Complutensis* **36**: 51–77.

Roth I. **1984**. *Stratification of tropical forests as seen in leaf structure*. Dordrecht ,the Netherlands: Distribution Center PO Box 322 3300 AH Kluwer Academie Publishers Group.

Russo SE, Cannon WL, Elowsky C, Tan S, Davies SJ. **2010**. Variation in leaf stomatal traits of 28 tree species in relation to gas exchange along an edaphic gradient in a Bornean rain forest. *American Journal of Botany* **97**: 1109–1120.

Rutter JC, Willmer CM. **1979**. A light and electron microscopy study of the epidermis of *Paphiopedilum* spp. with emphasis on stomatal ultrastructure. *Plant, Cell & Environment* **2**: 211–219.

Sha Valli Khan PS, Evers D, Hausman JF. **1999**. Stomatal characteristics and water relations of *in vitro* grown *Quercus robur* NL 100 in relation to acclimatization. *Silvae genetica* **48**: 83–87.

Stenström A, Jónsdóttir IS, Augner M. **2002**. Genetic and environmental effects on morphology in clonal sedges in the Eurasian Arctic. *American Journal of Botany* **89**: 1410–1421.

Tanner EVJ, Kapos V. **1982**. Leaf structure of Jamaican upper montane rain-forest trees. *Biotropica* **14**: 16–24.

Taylor SH, Franks PJ, Hulme SP, Spriggs E, Christin PA, Edwards EJ, Woodward FI, Osborne CP. **2012**. Photosynthetic pathway and ecological adaptation explain stomatal trait diversity amongst grasses. *New Phytologist* **193**: 387–396.

Tiwari SP, Kumar P, Yadav D, Chauhan DK. **2013**. Comparative morphological, epidermal, and anatomical studies of *Pinus roxburghii* needles at different altitudes in the North-West Indian Himalayas. *Turkish Journal of Botany* **37**: 65–73.

Toral M, Manríquez A, Navarro-Cerrillo R, Tersi D, Naulin P. **2010**. Características de los estomas, densidad e índice estomático en secuoya (*Sequoia sempervirens*) y su variación en diferentes plantaciones de Chile. *Bosque (Valdivia)* **31**: 157–164.

Vygodskaya NN, Milyukova I, Varlagin A, Tatarinov F, Sogachev A, Kobak KI, Desyatkin R, Bauer G, Hollinger DY, Kelliher FM *et al.* 1997. Leaf conductance and CO_2 assimilation of *Larix gmelinii* growing in an eastern Siberian boreal forest. *Tree Physiology* **17**: 607–615.
Wagner F, Below R, Klerk PD, Dilcher DL, Joosten H, Kürschner WM, Visscher H. **1996**. A natural experiment on plant acclimation: lifetime stomatal frequency response of an individual tree to annual atmospheric CO_2 increase. *Proceedings of the National Academy of Sciences, USA* **93**: 11705–11708.
Wagner F, Neuvonen S, Kürschner WM, Visscher H. **2000**. The influence of hybridization on epidermal properties of birch species and the consequences for palaeoclimatic interpretations. *Plant Ecology* **148**: 61–69.
Wang R, Yu G, He N, Wang Q, Xia F, Zhao N, Xu Z, Ge J. **2014**. Elevation-related variation in leaf stomatal traits as a function of plant functional type: evidence from Changbai Mountain, China. *PLoS ONE* **9**: e115395.
Zhang L, Niu H, Wang S, Zhu X, Luo C, Li Y, Zhao X. **2012**. Gene or environment? Species-specific control of stomatal density and length. *Ecology and Evolution* **2**: 1065–1070.
Zhang S-B, Sun M, Cao K-F, Hu H, Zhang J-L. **2014**. Leaf photosynthetic rate of tropical ferns is evolutionarily linked to water transport capacity. *PLoS ONE* **9**: e84682.
