# Supplementary figures and images for: Optimal allocation of leaf epidermal area for gas exchange
Source: New Phytol. 2016 Mar 16;210(4):1219–28. doi: 10.1111/nph.13929 (PMC5069575; doi:10.1111/nph.13929)

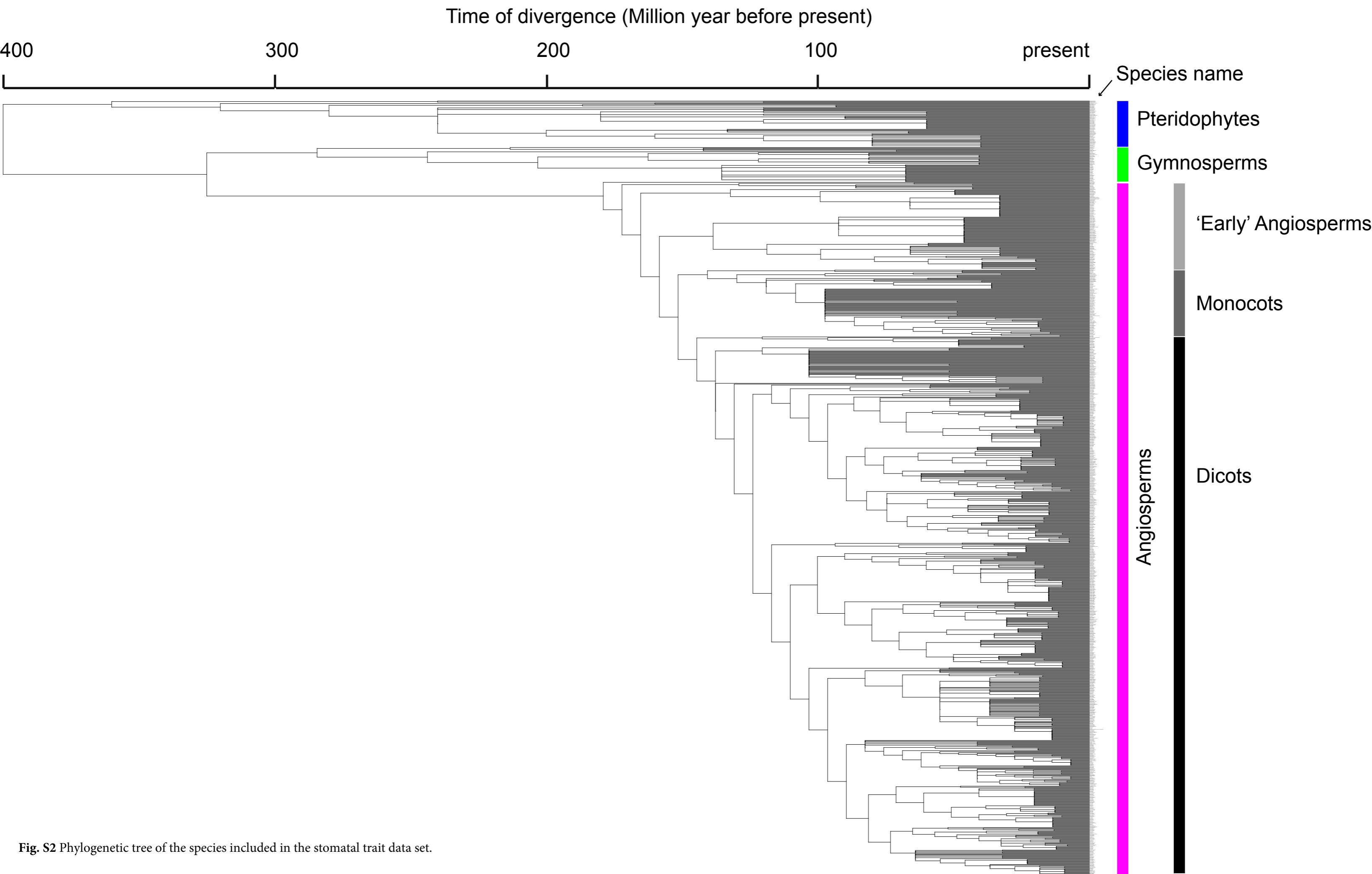

Fig. S2 Phylogenetic tree of the species included in the stomatal trait data set.

Supplement: Supplementary file 2 — Fig. S2 Phylogenetic tree of all species included in the stomatal trait dataset. [file NPH-210-1219-s002.pdf]
